# Growing PET positive nodule in a patient with histoplasmosis: case report

**DOI:** 10.1186/1749-8090-1-23

**Published:** 2006-09-04

**Authors:** Khaled F Salhab, Daniel Baram, Thomas V Bilfinger

**Affiliations:** 1 Department of Surgery, Stony Brook University Medical Center, Stony Brook, New York, USA; 2 Department of Medicine, Stony Brook University Medical Center, Stony Brook, New York, USA

## Abstract

**Background:**

Pulmonary histoplasmosis is a mycotic infection that often resembles pulmonary malignancy and continues to complicate the evaluation of pulmonary nodules.

**Case presentation:**

We report a case of an immunocompetent patient who, despite adequate treatment for known histoplasmosis lung infection, presented with radiological and F-18 fluorodeoxyglucose (FDG) positron emission tomography (PET) findings mimicking primary lung malignancy which eventually required surgical resection.

**Conclusion:**

Histoplasmosis infection may radiologically resemble pulmonary malignancy, often causing a diagnostic dilemma. PET imaging is currently used for and considered accurate in the evaluation of pulmonary nodules. However, overlap in PET standardized uptake value (SUV) between granulomatous and malignant lesions decreases the accuracy of PET as a diagnostic modality. Future advances in PET imaging are needed to improve its accuracy in the evaluation of pulmonary nodules in areas where histoplasmosis is endemic.

## Background

Histoplasma capsulatum is a dimorphic fungus that is usually found in the mycelial form in its native state and grows as a yeast at mammalian body temperatures. It was historically first described by Dr. Samuel Darling in 1906, using autopsy material obtained from a patient native to the island of Martinique that died from a wasting febrile illness. The name *Histoplasma capsulatum *was given to this organism since it was found to be present in histiocytes, and was thought to be an encapsulated plasmodium or protozoan. It has since been well established that *H. capsulatum *is not a plasmodium, but rather a dimorphic fungus that is usually found in the soil of endemic areas in many parts of the world.

The clinical and radiological presentation of pulmonary histoplasmosis is variable. Often, the infection may mimic lung neoplasm and may require thoracoscopy or open lung biopsy to establish a definitive diagnosis. F-18 FDG PET, increasingly used in the assessment of pulmonary nodules, is reevaluated as an accurate imaging modality in the diagnosis of pulmonary histoplasmosis in this case presentation.

## Case presentation

A 64-year-old fisherman presented to his primary care physician with fever, chills, and generalized malaise of three days duration. The patient had recently returned from a 2 week vacation to Guatemala and Belize. His medical history included prostate cancer and hyperlipidemia. He reported a distant history of tobacco-smoking. The patient appeared well and was in no acute distress. His physical examination was unremarkable except for an elevated temperature of 102 degrees Fahrenheit. Blood tests including complete blood count and differential, thick and thin smears for malaria, and dengue serology were normal. He was treated with oral clarithromycin 500 mg twice a day for one week. A second course of clarithromycin for a period of 14 days was initiated two weeks later for persistent fever and malaise, in addition to new complaints of severe bone pain and weight loss. After six weeks, the fever subsided and his constitutional symptoms improved.

**Figure 1:**
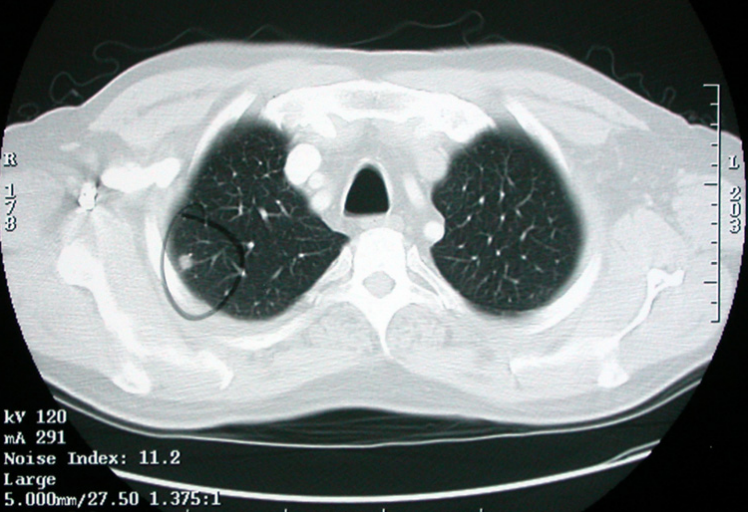
Chest CT scan showing a 1 cm right upper lobe nodule.

A Computed tomography (CT) scan performed during his fever evaluation revealed 2 pulmonary nodules: a 1.7 cm left lower lobe nodule and a 1 cm right upper lobe nodule (Figure 1). PET imaging revealed F-18 FDG uptake in the left lower lobe nodule with an SUV of 3.8, CT-guided needle biopsy was non-diagnostic, and the patient was referred to our hospital for further evaluation of suspected lung cancer.

He underwent a right thoracoscopy with wedge resection of the right upper lobe peripheral pulmonary nodule. Histopathology revealed multiple granulomata with extensive necrosis. Methenamine silver stains demonstrated yeast consistent with histoplasma within the necrotic foci. The patient was started on oral itraconazole capsules, 200 mg once a day.

**Figure 2:**
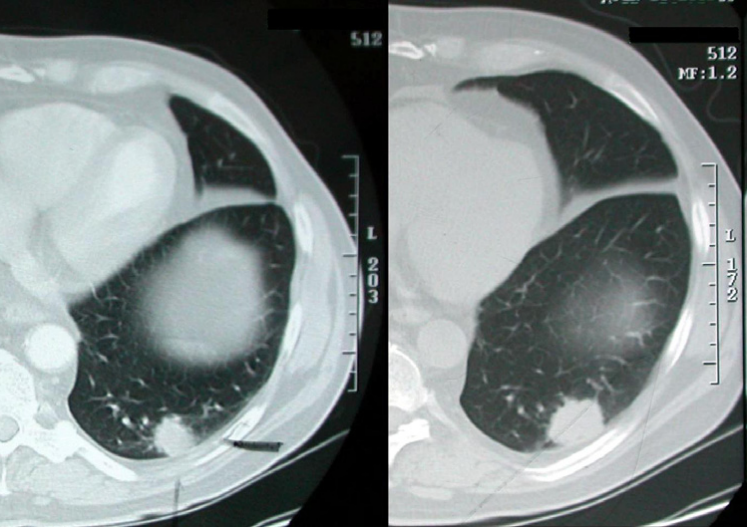
Chest CT scan showing a 1.7 cm left lower lobe nodule (*left*), as well as, a three months follow up Chest CT scan demonstrating enlargement of the left lower lobe nodule from 1.7 cm to 3 cm (*right*).

After three months, a follow up CT scan confirmed excision of the right lung nodule but demonstrated enlargement of the left sided lung nodule from 1.7 cm to 3 cm (Figure 2). Growth in a nodule with F-18 FDG uptake raised concern regarding cancer despite the known history of pulmonary histoplasmosis. The risks and benefits of operative resection versus watchful waiting were discussed with an interdisciplinary team, the patient, and his family.

The patient underwent a left lower lobectomy. Pathology revealed necrotizing granulomata resembling that seen in the prior biopsy; visible fungal elements consistent with histoplasmosis were visible upon silver staining. Interestingly, operative fungal cultures were negative, suggesting the growth in the lesion was likely from host immune response, not from progression of infection. The formulation of the patient's itraconazole was changed to an oral solution, 200 mg once a day for an additional three months. A follow up CT scan at one year duration has not shown any recurrence of disease.

## Discussion

*H. capsulatum *is endemic to many parts of the world, in particular Latin America and the areas of the Midwestern and Southcentral United States [1,2]. It is found to prefer moist soil as a habitat, mainly that contaminated by droppings of birds and bats [3,4]. The pathogenesis of histoplasmosis infection starts with the disruption of the soil. As a result, the hyphae and conidia of *H. capsulatum *become aerosolized, inhaled and deposited into the lung. There, the fungus is engulfed by macrophages and other phagosomes, but eludes elimination by converting to the yeast form. The yeast cells then reside and multiply within the phagolysosomal vesicles, possibly through a mechanism that elevates intraphagosomal pH [5,6]. In the absence of cell mediated immunity (CMI), the yeasts continue to multiply and disseminate to involve organs of the reticulo-endothelial system. Once CMI develops, in the immunocompetent adult, the formation of a granuloma begins and the intracellular yeasts are finally destroyed.

The clinical presentation of acute histoplasmosis usually depends on the size of the inoculum and the immune response of the host. The vast majority of infections are asymptomatic or present as an influenza-like illness, with cough, fever, and headache. Chest radiographic findings typically demonstrate small areas of infiltrate or mediastinal lymphadenopathy. With time, a granulomatous reaction occurs with areas of caseating necrosis leading to the appearance of a round mass of scar tissue that may or may not be calcified, known as a histoplasmoma.

In patients with known underlying lung disease, such as emphysema or COPD, a chronic form of pulmonary histoplasmosis may occur. This infection usually starts by the colonization of diseased lung tissue in the upper zones, and progresses gradually, leading to cavity formation and scarring. Symptoms are characterized by a productive cough, fever, hemoptysis, weight loss, and occasional night sweats along with lung cavitations that may be seen on chest radiographs, often mimicking tuberculosis.

Patients with inadequate CMI, such as those receiving immunosuppressive therapy or those infected with the Human Immunodeficiency Virus (HIV), may develop progressive disseminated histoplasmosis, an often rapidly progressive and fatal illness that presents with fever, anemia, leukopenia, hepatosplenomegaly, central nervous system involvement, and cardiac manifestations.

Several laboratory tests are useful in the diagnosis of infections caused by *H. capsulatum*. Culture samples of the organism remain the gold standard for diagnosis. The samples are usually recovered from bone marrow, liver, sputum, and bronchoalveolar lavage. Histopathologic visualization using special stains such as Giemsa or Methenamine Silver stains also aids in the diagnosis and yields very rapid results. Complement fixation is another test used for the diagnosis of *H. capsulatum*, detecting both mycelial and yeast antigens.

Most patients with the acute form of histoplasmosis do not require antimicrobial therapy, as they remain asymptomatic or improve spontaneously. Patients presenting with the chronic form of histoplasmosis are usually treated with itraconazole for 6–12 months as a first line of treatment. Patients presenting with progressive disseminated histoplasmosis usually require induction therapy with intravenous amphotericin B, although it has been shown that some patients may respond to itraconazole as a first line therapy [7]. Once stabilized, patients may be switched from amphotericin B to itraconazole. Patients with HIV who present with progressive disseminated histoplasmosis require life-long maintenance therapy.

Surgical intervention is occasionally required in patients with histoplasmosis. The finding of a histoplasmoma presenting as a lung nodule or mass on a chest X-ray or CT scan often raises concern for malignancy, which may require thoracoscopic or open lung biopsy to establish a definitive diagnosis.

F-18 FDG PET imaging and semiquantitative analysis with SUV is increasingly used in the work-up of pulmonary nodules [8]. Though generally considered accurate and clinically useful, there is considerable overlap in SUV values obtained from active granulomatous lesions and malignant processes [9,10]. Most reports of PET imaging in granulomatous disease are of mycobacterial infection or sarcoidosis; review of the literature reveals scant information on FDG-PET and SUV values pertaining to histoplasmosis [11-14]. In fact, this information is based on the published experience of 7 patients only (Table 1).

**Table 1:** Standard Uptake Values of FDG-PET in Histoplasmosis

Author	No. of Patients	SUV	Pathology
Gupta, 1992	2	0.42 and 0.82	Histoplasmosis
Dewan, 1993	2	1.92 and 3.38	Caseating granuloma with Histoplasma
Lowe, 1998	1	5	Necrotizing granuloma with histoplasmosis
Croft, 2002	2	4.0 and 4.5	Histoplasmosis

This case illustrates the difficulty in differentiating active histoplasmosis infection from malignancy. Despite known pulmonary histoplasmosis infection, there remained significant concern that the left lower lobe lesion of the patient presented herein was a concurrent malignancy, especially when the lesion grew in spite of adequate antifungal therapy. An increase in FDG uptake, with an SUV of 3.8, further raised this concern. A second pulmonary resection was ultimately required to definitively rule out malignancy in this otherwise healthy, immunocompetent individual.

In conclusion, though a self-limited disease in most patients, *H. capsulatum *infection continues to complicate the evaluation of pulmonary nodules and often leads to surgical resection. PET imaging is likely not useful in determining the correct clinical approach in this disease, as false positives are quite common in granulomatous lesions. Perhaps future advances in PET imaging or the use of delayed scanning may increase the accuracy of this modality for cancer detection in areas where histoplasmosis is endemic.
